# Supplementary material for: Are carbon emissions trading and green financial instruments synergistic? -Comprehensive quantitative research based on content analysis
Source: PLoS One. 2024 Mar 7;19(3):e0298601. doi: 10.1371/journal.pone.0298601 (PMC10919601; doi:10.1371/journal.pone.0298601)
Supplement: S1 Data — (ZIP) [file pone.0298601.s001.zip › Data/地级市Z_F工作报告.zip!/┴╔─■/╡ñ╢1⁄2╩╨/└┤╫╘╣1⁄2╓┌║┼í╛┬φ┐╦╩2╛▌═°í┐_2012.docx]

各位代表：

　　我代表市人民政府向大会作政府工作报告，请予审议，并请市政协委员和各位列席人员提出意见。

　　一、2011年政府工作的回顾

　　2011年，在省委、省政府和丹东市委的正确领导下，在市人大、市政协的监督支持下，面对复杂严峻的国际经济形势、艰巨繁重的改革发展任务，市政府组织和带领全市人民攻坚克难，真抓实干，圆满完成年初确定的各项目标和任务，实现了经济社会平稳较快发展。预计2011年，全市地区生产总值实现905亿元，比上年增长17.1%;规模工业增加值310亿元，增长20%;固定资产投资700亿元，增长33%;地方财政一般预算收入实际完成103.3亿元，增长29%;预计实际利用外商直接投资突破10亿美元;外贸出口总额23亿美元，增长22.7%;引进内资项目到位资金750亿元，增长50%;全社会消费品零售总额325亿元，增长18%;城镇居民人均可支配收入16978元，增长16.8%;农民人均纯收入突破10000元。

　　——重大项目快速推进。新鸭绿江公路大桥开工建设，中朝两国合作开发朝鲜黄金坪正式启动并初步完成总体规划，特殊经济配套区正在积极组织申报和编制规划。基础设施建设中的沈丹客运专线和丹大快速铁路项目路基、桥隧涵等工程全线展开;丹通高速公路进入路面施工阶段，丹海高速公路建成通车，东北东部铁路初步具备通车条件;三湾水利枢纽工程快速推进，蒲石河抽水蓄能电站第一台机组实现发电;丹东港全年累计完成投资102亿元，吞吐量实现7550万吨，比上年增长36%，20万吨矿石码头项目获省批复;丹东机场新航站楼完成外装修工程。工业项目中，帕斯特谷物二期等一批项目开工建设;哈曼丹东制造基地、凤城翁泉沟硼化工基地一期工程等项目竣工投产;本钢不锈钢冷轧板、金山热电等项目进入设备安装调试阶段;造船业实现突破，丹东船舶重工两艘大型工程船建成下水。商贸旅游项目中，沃尔玛购物广场开业，凤凰山景区基础设施建设项目开工，宽甸天桥沟开发项目休闲区设施交付使用。

　　——沿江沿海经济带保持较快建设步伐。以新区、大孤山经济区为重点的沿江沿海经济带建设快速推进，重点园区初具规模，有力带动了全域开发。丹东新城区城市功能项目加快实施，新建市民服务中心全面启用，城市综合管理水平逐步提升，城市功能日渐完善，各类产业项目不断向园区集聚。东港经济区保持较快发展步伐，再生资源产业园国家“城市矿产”示范基地获得国家发改委、财政部批准。大孤山经济区海洋红港区西侧航道试挖槽开工，临港重化工、物流、精密等专业园区建设有序推进，中央大街、城市排水等城市改造工程顺利展开。沿江沿海经济带重点区域全年累计开工项目436个，完成投资额115.6亿元。

　　——工业经济实力进一步增强。预计规模以上工业主营业务收入完成1250亿元，比上年增长38%;税金42亿元，增长28%;规模以上工业企业突破900户，其中亿元以上企业达到256户，新增70户;个体工商户转企升级 2257户。工业固定资产投资预计完成400亿元，增长10.5%，占全市固定资产投资比重达57%。新开工亿元以上工业项目31个。新认定高新技术企业7户，高新技术产品增加值增长25%。10余户企业启动上市程序。以仪器仪表产业基地等重点园区为拉动，形成了超10亿元以上产业集群11个，主营业务收入预计达到810亿元，增长34%。

　　——农业、农村工作稳步推进。粮食产量再创历史新高，预计达到99.6万吨。新增种植业设施农业面积7050亩，新增标准化“三品”生产面积97万亩，水产品总产量达到55.3万吨，东港梭子蟹、黄蚬子、杂色蛤通过国家地理标志产品认证。建设畜禽养殖小区386个，肉蛋奶总产量46.8万吨。新增投资10亿元以上农产品加工重大项目4个，新增主业收入超亿元企业5户。东港草莓产业获全国草莓第一县和省政府“一县一业”认定。县域经济快速发展，东港市在全国百强县位次由上年的83位前移至75位，凤城市也首次进入全国百强县并位列第87位，宽甸综合实力实现提升。完成农建工程投资5.4亿元，大中型病险水库全部得到除险加固。植树造林18.4万亩，栽植树木6100万株，是历年来投入最大的一年。近11万农村人口饮水困难得以解决。渔业船舶在省内率先安装先进电子系统，“三无”渔船得到有效管控。积极争取“兴边富民行动”的政策支持。全面落实各项惠农政策，扶贫工作得到国家和省里高度评价。

　　——服务业发展速度不断提高。实施了9个现代服务业集聚区建设，其中中央商贸商务服务业集聚区被纳入首批省级现代服务业集聚区，列入省考核的3个服务业集聚区均完成了各项发展指标。汇侨购物广场、希尔顿酒店等一批项目相继落地并开工建设，带动了城市新商业圈的形成。实施了虎山、天桥沟、河口等旅游景区、景点改造工程，温泉旅游度假区、温泉旅游小镇及温泉旅游项目的规划、招商和建设快速推进。赴朝旅游集聚区被列入省重点建设的六大旅游集聚区项目。在央视等主流媒体的金牌栏目中开展旅游宣传，通过旅游合作使市场得到进一步拓展。启动第二批向城区下放管理权限工作，城区经济实现了第一个“三年倍增”目标。

　　——对外开放步伐持续加快。精心举办了中国·丹东鸭绿江国际旅游节、中国丹东仪器仪表博览会、辽宁台湾周等大型展会活动，赴温州、宁波等城市以及日本、韩国、台湾、香港等国家和地区开展主题招商，取得明显成效。积极落实重点外资项目。组织外贸出口企业参加广交会、朝鲜春秋季商品交易会、东盟博览会等国内外大型展销会，搭建企业出口服务平台，促进了外贸出口持续增长。与沈阳和大连的合作取得新进展，与东北东部城市在人力资源、旅游开发、陆港建设等方面合作迈出新步伐。

　　——财政金融稳步增长。财政收入结构逐步优化，收入质量进一步提高。用于民生方面的新增支出占支出增量的80%。国有资产监管得到加强。预计全市金融机构各项存款余额突破1000亿元，贷款余额接近600亿元，存贷比提高了4个百分点。浦发银行等金融企业在我市设立分支机构工作得到积极推进。小额贷款公司、村镇银行、担保公司等新型金融机构累计贷款余额45亿元。银行贷款之外的融资渠道不断拓宽，融资总额达到120亿元。

　　——各项民生实事有效落实。提前谋划供暖工作，筹措供暖保障资金6500万元，供暖期开始前全市储煤率达到80%以上，确保了按时开栓和百姓温暖过冬，居民关于供暖的投诉量大幅下降。累计投资26.7亿元，实施保障性安居工程建设，国有工矿棚户区改造，林区、垦区危房改造共计9050套已全部交付使用，新建廉租住房、公共租赁住房、经济适用住房1841套，城市棚户区改造10887套，开工率超额完成省政府要求的指标。实施城建项目136个，其中“小巷工程”完成76条居民小巷改造，极大方便了市民出行。滨江中路景观改造大部分完工，城市新增公园和绿地167公顷，城市生态环境进一步改善。基本完成增殖放流、城市燃气储罐改造、供水和新区安居工程等15件惠民实事，涉及群众生活的热点难点问题逐步得到解决。采取科学有力的措施，既保持了房地产业健康发展，又有效遏制了房价过快增长势头，全年房价涨幅控制在9.5%以内。新增城镇就业6万人，城镇登记失业率控制在4.2%以内。启动实施了新型农村和城镇居民社会养老保险工作，60周岁以上农村居民19.6万人和城镇居民1.6万人享受了养老金待遇，实现了社会保障的全覆盖。落实对困难群体的救助政策，城乡低保、农村五保、重点优抚对象和孤儿的生活保障标准进一步提高，4所农村困难家庭常年病人托管中心建成并投入使用，兴建了一批村级公益项目。积极、科学而有效地应对炭疽疫情，在短时间内予以控制和解除。提前准备，周密安排，精心做好“米雷”、“梅花”两次强台风的预防和应对工作，确保了人民群众的生命财产安全。

　　——社会事业进一步繁荣。全面完成义务教育“双高”普九任务，投入1.9亿元用于学校基础设施建设，学校办学条件明显改善，标准化农村九年一贯制学校达到30 所，5所职业技术学校列入国家重点职业学校。投入2亿元，完成卫生基础设施建设6.8万平方米，政府办基层医疗机构全部实施了基本药物制度。群众性体育活动深入开展，广场文化、社区文化进一步丰富，文化体制改革稳步推进。成功举办了“鸭绿江之夏”人才招聘会和“鸭绿江之友”院士专家联谊会活动，引进国外专家项目27个。“四城联创”活动取得阶段性成果，通过了国家园林城市实地验收，获得了省级卫生城市称号，环境保护和生态建设取得新进展。双拥工作成果显著，有望荣获全国双拥模范城“六连冠”。进一步强化了食品药品监管，对小餐饮等社会热点问题进行了重点整治。深入开展了安全生产专项整治行动，全年无重大以上事故发生。认真解决群众合理诉求，信访积案化解取得实效。政府廉政建设不断加强，软环境建设继续优化，民主法制、社会治安管控等工作扎实推进，应急管理体系进一步完善，边海防管理水平有效提升，社会主义民族关系得到巩固，保持了社会和谐和边境稳定。主动接受市人大的法律监督、工作监督和市政协的民主监督，认真办理人大代表建议和政协提案514件，办结率和满意率均为100%。

　　外事、侨务、口岸、台务、宗教、统计、物价、人口计生、保密、档案、地方志、接待、人防、气象、防震减灾等工作也取得了新成绩。这些成绩的取得，是省委、省政府和市委正确领导的结果，是市人大、市政协大力支持的结果，是全市上下共同奋斗的结果。在此，我代表市政府，向全市人民、各条战线广大干部职工、驻丹部队官兵表示衷心的感谢并致以崇高的敬意!

　　回顾过去，成绩令人欣慰，但我们也清醒地看到，我市经济社会发展还存在一些困难和问题，突出表现在：一是经济总量偏小的问题一直没有实质性改变，缺乏对地区经济具有较强牵动和支撑作用的大企业、大项目;二是产业结构不尽合理，高端项目少、高附加值产品少;三是财政困难状况仍未根本缓解，社会保障难度加大;四是投资软环境有待进一步改善，机关作风不实、审批效率不高的现象仍不同程度存在。对此，我们将高度重视，采取更加符合发展实际和针对性强的措施，努力加以改进。

　　二、2012年经济和社会发展的主要任务

　　2012年是本届政府的收官之年，是确保完成本届政府所确定各项目标任务的关键一年。我们看到，当前世界经济增长放缓，各类风险明显增多，国内经济增长下行压力增强，经济形势十分复杂严峻，这些不稳定性、不确定性影响着我们对总体形势的把握。但我们也应看到，国际投资的热点已经转向我国沿海一线，随着辽宁沿海经济带国家战略的深入实施，我市仍处在重要的发展战略机遇期，加之丹东业已形成的良好发展态势，使我们具备了进一步加快发展的基础。因此，我们必须科学判断、理性分析、积极应对面临的形势，有效利用具有的优势和条件，进一步明确符合丹东实际的发展目标和方向，按照中央和省委、省政府提出的稳中求进、稳中求快的要求，把握机遇，迎难而上，努力实现高质量、快速度发展。为此，今年政府工作的指导思想是：以邓小平理论和“三个代表”重要思想为指导，深入贯彻落实科学发展观，认真落实中央经济工作会议，省委、市委十一届二次全会暨经济工作会议精神，以开放为主基调，以发展为推动力，以促进人民生活水平和质量持续提高为着眼点，进一步优化产业结构，使农业发展更稳、工业发展更强、服务业发展更快，提升发展质量，促进生态和谐，把改革的成果更多地惠及于民，着力建设务实、负责政府，努力打造大气秀美新丹东。

　　2012年全市经济和社会发展的主要预期目标为，地区生产总值增长15%，规模工业增加值增长18%，固定资产投资增长30%，地方财政一般预算收入增长18%，实际利用外商直接投资完成10亿美元，外贸出口总值增长18%，引进内资项目到位资金增长30%，全社会消费品零售总额增长17%，城镇居民人均可支配收入增长14%，农民人均纯收入增长13%。为实现上述目标，今年要全力抓好以下重点工作：

　　(一)坚定不移地加快沿江沿海开发开放步伐

　　抓好重大项目落地。把项目落地作为今年工作的重点，完善基础设施建设，千方百计解决好土地动迁、收储、要件审批等各个环节中的问题，特别是对已签约且符合丹东产业发展方向的重大项目，认真梳理影响落地的因素，列出落地时间表，明确责任，跟踪服务，提高审批效率，使落在纸面的项目真正落到地上。督促项目提高投资强度，采取措施防止围而不建问题，使开工项目加快建设速度，缩短建设工期，尽快竣工达产。今年要实现丹通高速公路、东北东部铁路通灌段竣工通车，丹东机场新航站楼投入使用，蒲石河抽水蓄能电站、金山热电、本钢不锈钢冷轧板等重点项目正式投产。

　　加快新区建设。以商贸、会展、餐饮娱乐等现代服务业为龙头，全面带动新区各类产业发展，不断完善城市功能，力争国门湾金融会展城等项目开工，国门湾家居广场等项目尽快投入运营，确保竣工运营面积100万平方米以上，逐步实现新区繁荣。以聚集人流、提升人气为重点，做精做细新区城市功能项目，吸引居民入住，确保新区大剧院、规划馆及体育馆、游泳馆、训练馆竣工投入使用，同时，加快新区行政、教育、文化、卫生、交通、公安、通讯等各类功能性项目和体系建设，推进新区向新城迈进。以专业园区为载体，着力引进高技术含量、高投资强度、高税收贡献率的大项目，重点打造仪器仪表及视光产业、IT软件产业、不锈钢产业、高端音响及电子产业、纺织产业、船舶制造产业6个集群，其中仪器仪表产业基地争取250—350户企业入驻。整体开发建设中朝黄金坪特殊经济配套区，做好申报工作，积极争取国家在海关监管、土地利用、零售免税、劳务用工以及支持边境经济发展方面的特殊优惠政策。充分开发和利用好新区土地资源，实现高效开发、集约利用，使项目和园区建设得到有力保障。加快安民山周边地区改造，做好新区保障性住房建设。

　　启动“翡翠湾”开发建设。高标准规划、高起点启动开发江湾地区22平方公里区域，打造新老城区连接带，形成“商业金廊”和“生态走廊”，逐步建设高端、高档的现代服务业集聚区和居住社区。启动建设城市四号干线，实行分期实施，使城市南北方向实现拓展，并以此改造和开发城市北部地区。

　　全面推进大孤山经济区建设。进一步完善城市基础设施，确保完成主干路网骨架。全力展开重点园区、新城区建设，力争临港产业园起步区完成征地动迁和安置房建设，精铸工业园等初步具备企业入驻条件。

　　大力发展港航经济。全力抓好丹东港建设，不断完善港口功能和配套设施建设，提高港口生产自动化、管理专业化和信息化水平，特别是把发展集装箱运输放在突出位置，提升港口吞吐能力，向亿吨港迈进。积极推进海洋红港各项审批和征海征地等工作。依托港口拉动，加快临港、临海产业项目的引进和落地，做好物流业的规划和发展，逐步形成临港产业链条。

　　实现引资向选资转变。今年招商引资的重点是引进具有高投资强度、高科技含量、高附加值项目和战略性新兴产业项目，污染项目绝不能进入丹东，保护好丹东的青山绿水。招商引资的主攻方向为大型国有企业、世界500强企业、行业百强企业，保证引进项目的质量和规模。精心举办好中国(丹东)中朝经贸文化博览会，力求办成一个新的会展品牌。以对朝贸易为重点，拓展对外贸易渠道，扶持外贸出口骨干企业，稳定出口、扩大进口。进一步加强与东北东部地区，沈阳、大连等中心城市的区域合作。

　　(二)增强工业经济支撑作用

　　支持重点骨干企业做大做强。大力实施择优扶强战略，以百户企业为重点，研究制定扶持政策，加大财政支持力度，重点协调解决企业融资难、招工难、用地难等瓶颈问题，促进企业升级改造。今年市财政将在原有基础上，再增加1000万元专项资金，助推和扶持骨干企业实施技术改造，加快现有产品更新换代，增加产品附加值。同时，扶持和壮大本地成长型企业，重视和支持中小企业发展。

　　突出抓好产业集群发展和园区建设。立足资源优势和产业基础，积极培育具有地区特点的千亿元产业集群和百亿元工业园区。以高端装备制造业特别是精密仪器仪表为重点，着力引进有品牌、有影响、有牵动力的工业大项目。抓紧制定产业集群发展规划，落实相关鼓励和扶持措施，重点抓好汽车及零部件、仪器仪表、精密装备、电子信息、农产品加工、纺织服装、硼铁硼镁资源深加工等重点产业集群。加快推进重点园区基础设施建设和功能配套，充分发挥工业园区的载体作用，引导企业和项目向园区集聚。

　　提升企业创新能力。以优化科技创新环境、提升科技创新能力为重点，推进高新技术和新兴产业发展项目，开展15项重大科技攻关。新组建产业技术创新战略联盟3个，实施重点产学研合作项目15项，特别是深化同中科院的全面合作。推广转化15项优秀科技成果和专利技术，新建各级各类企业工程技术研究中心10户。注重知识产权保护，争创著名商标和知名品牌。

　　强化工业经济运行。密切关注宏观政策和市场变化对工业发展的影响，加强对经济运行的调度和监测分析，积极引导企业正确判断市场走势，及时掌握生产要素供求变化情况，提高市场应变能力，主动抢占市场。强化煤、电、油、运等要素的协调平衡，主动协调解决企业生产经营中的实际问题。

　　(三)推动县域经济快速发展

　　扎实推进工业化建设。高度重视大项目引进和建设，特别是抓好10亿元农业项目的尽快落地、建设和投产，提升重点工业园区的产能和效益。突出区域特色，加快东港市再生资源产业园、凤城市辽东硼铁资源综合利用产业基地、宽甸硼铁硼镁精深加工产业集群建设，力争新增3-5家主业收入超亿元企业。

　　做大做强现代农业。稳定粮食生产，推进设施农业建设，发展“一县一业”，抓好特色产业基地建设，加大农业新品种、新技术的研究和推广力度，大力发展有机、绿色、无公害产品。着力抓好蔬菜生产基地建设，提高地产蔬菜特别是无公害蔬菜的市场供应量。积极培育龙头企业，新增主业收入亿元以上农产品加工企业5户。产业化示范区力争进入省级示范区3个、国家级示范区1个。

　　改善农村生产生活条件。加大农村基础设施建设力度，抓好小城镇建设，促进农村人口向城镇转移。加强农田水利建设，加快三湾水利枢纽工程实施。注重耕地保护和水土保持，推进青山保护工程。抓好水源和节水灌溉工程建设，解决8万农村人口饮水困难问题。积极推进农村路网建设，实施村容镇貌综合整治，推广农村新能源、新技术，改善农村生态环境。加强农村集体资产、资源、资金管理，落实各项惠农政策，推广应用农业新技术，努力打造全国“兴边富民行动”示范区。

　　(四)提升服务业发展水平

　　加快服务业集聚区建设。从业态提升、水平提高入手，优化城区产业结构，合理规划服务业布局，大力发展信息软件、科技服务、现代物流、金融服务、商务服务、文化创意等现代服务业集聚区，积极发展总部经济和会展经济，增强城区经济发展活力。加快推进万达城市广场、鹏欣水游城、希尔顿酒店、汇侨购物广场、红星美凯龙商业广场等大型服务业项目建设，提升商贸服务业集聚区规模档次。着力打造朝鲜族餐饮服务集聚区。以五龙国际服务外包产业园为重点，加快发展科技服务业集聚区。进一步活跃新区商业氛围，促进东港、凤城、宽甸服务业集聚区发展。

　　增强旅游产业牵动力和产品吸引力。充分发挥丹东青山绿水优势，加快鸭绿江、凤凰山、天桥沟等景区、景点的深度开发，提升景区的档次和水平。鸭绿江风景区积极争创国家5A级景区，打造鸭绿江精品旅游带。做精四季旅游产品，丰富旅游文化内涵，培育和推出精品旅游线路。抓好温泉资源开发利用和温泉旅游项目规划，积极申报国家“温泉之城”，推进一批大投入、高档次的温泉旅游项目。继续加大赴朝旅游集聚区项目建设力度，大力发展边境旅游、乡村旅游、海岛旅游，开发休闲旅游产品。拓展营销新渠道，扩大丹东旅游宣传覆盖面和影响力。注重引进和建设旅游星级酒店，提高旅游接待能力和服务水平。

　　(五)强化财政金融保障

　　加大财政保障力度。积极培植税源，继续降低非税收入比重，不断提高财政收入质量，增强财政可支配能力。做好土地整理，认真抓好土地出让。从严控制财政一般性支出，大力节约行政开支，能不买的坚决不买，可不支的坚决不支，集中更多的财力用在民生方面。加强政府债务管理，切实防范财政风险，做好对资金使用的审计，强化国有资产运营。

　　积极做好金融融资。引进银行机构2-3家，引导银行机构增加贷款投放;新增小额贷款公司6家，发挥好小额贷款公司、担保公司作用。鼓励帮助企业上市融资，年内确保1户、力争实现2户企业上市。力争实现各类融资230亿元。

　　(六)全力做好民生工作

　　办好惠民实事。今年，我们将在涉及百姓生产生活最直接的方面，拿出一定资金，为群众做好15件具体实事。1. 机关联系企业帮扶135个贫困村，解决8万困难和相对困难农民的收入问题;2.在近郊开工建设一处大型蔬菜批发市场，缓解菜农卖菜难和居民买菜贵问题;3.在15家超市和农贸市场建立蔬菜、水产品质量监测中心，配备蔬菜、水产品检测仪器，方便群众监测，保证食品安全;4.启动金山污水处理厂建设，解决企业和居民污水排放问题;5.建设保障性住房5820套;6.实施煤气设施改造，更换老旧煤气表1万台，对1万户室内腐蚀管网进行更换维修，增强市民用气的安全性;7.对全市4000余个垃圾楼道全部实施封堵，彻底解决困扰几十年、涉及30万居民的楼道环境问题;8.实施城区旱厕改水厕工程;9.实施“小巷工程”，完成66条街道小巷改造;10. 在23处易发事故和易堵塞的交通路口新建交通信号灯，在主要街路新增192处交通电子监控点;11.在新老城区公共场所设置300个高清视频探头，加强社会治安监控，增强百姓安全感;12.投放100辆清洁能源公交车，并增加新区公交车辆及班次，使市民出行更便利;13.新建20所乡镇以上公办幼儿园，进一步缓解入园难问题;14.启动中心医院扩建工程;15.开播电视手语节目，为残疾人服务。

　　加强城市环境治理。扎实推进“四城联创”，以建设环境良好、干净整洁的城市为目标，抓好生态治理，严把项目的环境准入关，从源头预防污染，加大环境执法力度，强化对重点污染源、减排重点行业和高污染、高耗能企业的监管，对于污染严重的企业，逐步实施搬迁或转产。继续对大沙河、花园河等城市内河进行综合治理，加快城市公园建设，对城市道路、广场、空地、内河两岸按照城市规划精心做好绿化美化。加强市容环境卫生综合整治，实施旧小区、老旧建筑改造，对部分公共建筑和主要街路楼体做好亮化。抓好二号干线、花园东路、东泰路等部分主干路的贯通、拓宽工程建设，完成城市30条道路的大修。积极推进金山热电供热联网，加强供暖监管，提高供暖质量，为群众提供温暖、舒适的生活环境。

　　强化有序的管理体系。治理乱开发问题，科学做好城市规划并严而有序地实施建设改造。继续治理乱开采问题，完善资源开采规划，加强对矿产、温泉、砂石、地表水及地下水、山林等资源的监管，提高资源利用率。做好土地储备，加大闲置土地清理。强化交通管理和整治，优化调整城市公交线网，科学设计交通线路，合理布局停车场，加强各类车辆管理，保持顺畅的交通秩序。规范市场秩序，有效治理占道经营和马路市场扰民问题。加强价格监管，稳定市场物价。

　　促进就业和社会保障工作。扩大就业渠道，缓解“就业难”，重点加大大中专毕业生和城镇失业人员的就业力度。做好养老、失业、医疗、工伤、生育等保险扩面和新农保、城居保工作。加强失地农民生活保障。推进社会救助精细化管理，完善城乡低保、农村五保、医疗救助、供暖救助、廉租房补贴分配等工作机制，全力保障困难群众基本生活。

　　(七)积极创新社会管理

　　繁荣社会各项事业。启动基础教育强县(市)区创建工作，加强农村基础教育，着力抓好与丹东产业发展相衔接的职业教育。努力提高基本医疗服务能力，促进公共卫生服务均等化。深入开展“文明使者在行动”等城乡群众性精神文明创建活动。围绕大连、丹东沿海沿江文化创意产业先导区建设，进一步深化文化体制改革，加快发展文化产业，培育丹东特色文化品牌，争取大孤山文化产业园列入省重点文化园区。加强公共文化服务体系建设，实施文化惠民工程，广泛开展群众性文化、体育和全民健身等活动。积极引进高层次人才，注重培养本地区各类专业人才。做实国防动员工作，加强民兵预备役建设，创新双拥工作模式和内容。落实人口计生政策，切实加强社区建设，做好社会养老服务等工作。

　　营造平安和谐的社会环境。搞好普法宣传教育。依靠科技强警和信息化建设，完善防控体系，严厉打击各种违法犯罪行为，为群众创造一个安全的社会环境。强化边海防管理，加强边海防整治，结合边境的特点，严厉打击走私、贩毒等犯罪活动，重视民族宗教工作，维护国家安全，确保边境稳定。全面推进领导干部大接访和领导包案工作，推动矛盾纠纷化解。深入开展食品药品领域的监督检查和集中整治，加大对食品药品领域违法犯罪的打击力度，在食品药品的生产、流通、经营等各个环节强化监管，让老百姓放心饮食、安全用药。高度重视安全生产，强化责任落实，确保不发生重大以上安全生产事故。加强应急管理，不断提高政府保障公共安全和处置突发事件的能力。

　　(八)努力建设务实、负责的服务型政府

　　转变工作作风。树立百姓的诉求就是对政府的要求理念，认真解决群众反映强烈的各类问题，为丹东百姓做事，对丹东百姓负责，把群众的事作为政府的责任，不折不扣、千方百计去推进和落实。建设务实政府，少说多做抓落实，力戒虚夸和浮躁，对做事摆花架子、搞形式主义、揽功诿过的部门和人员坚决问责和处理。政府各级领导干部要多拿出一些时间到基层和群众中去调研，提倡现场办公，为群众的生活和企业的生产经营排忧解难，解决具体问题。

　　严格依法行政。提高政府工作人员特别是各级领导干部依法行政意识和水平，自觉接受人大法律监督、工作监督和政协民主监督，坚持向人大报告工作、向政协通报工作制度，主动听取社会各界的意见和建议，认真落实人大及其常委会的决议、决定，切实做好人大代表建议和政协提案的办理。更加注重社情民意调查，完善重大行政决策听证、公开征求意见等制度，进一步畅通群众诉求和沟通渠道，更好地听民声、解民忧。加大对决策失误、责任事故、行政不作为等的问责力度，抓好诚信建设，增强政府公信力。

　　提高服务效率。加强公务员队伍管理，有针对性地组织专题培训，强化公务员能力素质建设。深化行政审批制度改革，推进政务服务体系和电子政务建设。抓好部门窗口服务质量，解决服务态度冷漠、办事拖拉等问题，对在群众办事、企业项目审批等过程中出现的吃拿卡要，不给钱不办事、给了钱乱办事等现象进行严肃查处，为群众提供一个舒心、顺畅的服务环境。深化政务公开，推动政府信息公开向基层延伸。

　　加强廉政建设。推进惩防体系建设和“五大系统”建设，深化行政审批、财税、金融和投资体制改革以及国有资产管理体制改革，坚决刹住公务开支中奢侈浪费的不良风气。开展“小金库”等专项治理，坚持政风行风评议制度，严肃查处损害群众利益的不正之风和违法违纪问题。完善政府采购、工程招投标制度。充分发挥审计、监察功能，加强对行政权力运行、财政资金使用、经济责任履行等领域的监督。

　　各位代表，我们正处在新一轮扩大开放和加快发展的关键时期，在机遇和挑战面前，肩负的任务和使命艰巨而光荣。我们一定要在市委的坚强领导下，在市人大、市政协的大力支持下，凝聚全市人民的智慧和力量，振奋精神，万众一心，锐意进取，乘势而上，加快建设大气秀美新丹东，以更加优异的成绩向党的十八大献礼!
